# Regulation of gut microbiota: a novel pretreatment for complications in patients who have undergone kidney transplantation

**DOI:** 10.3389/fcimb.2023.1169500

**Published:** 2023-06-06

**Authors:** Jiajia Ye, Junxia Yao, Fangfang He, Jing Sun, Zheng Zhao, Yumei Wang

**Affiliations:** ^1^ Department of Nephrology, Union Hospital, Tongji Medical College, Huazhong University of Science and Technology, Wuhan, China; ^2^ Institute of Hematology, Union Hospital, Tongji Medical College, Huazhong University of Science and Technology, Wuhan, China

**Keywords:** kidney transplantation, gut microbiota, complication, diarrhea, rejection, probiotic

## Abstract

Kidney transplantation is an effective method to improve the condition of patients with end-stage renal disease. The gut microbiota significantly affects the immune system and can be used as an influencing factor to change the prognoses of patients who have undergone kidney transplantation. Recipients after kidney transplantation showed a lower abundance of Firmicutes and *Faecalibacterium prausnitzii* and a higher proportion of Bacteroidetes and Proteobacteria. After using prebiotics, synbiotics, and fecal microbiota transplantation to regulate the microbial community, the prognoses of patients who underwent kidney transplantation evidently improved. We aimed to determine the relationship between gut microbiota and various postoperative complications inpatients who have undergone kidney transplantation in recent years and to explore how gut microecology affects post-transplant complications. An in-depth understanding of the specific functions of gut microbiota and identification of the actual pathogenic flora during complications in patients undergoing kidney transplantation can help physicians develop strategies to restore the normal intestinal microbiome of transplant patients to maximize their survival and improve their quality of life.

## 1 Introduction

Kidney transplantation is among the most effective treatments for end-stage kidney diseases compared with conventional therapies. Kidney transplantation was first introduced in the 1950s (Meena et al., 2021), and since then, it has drastically improved the survival rates of patients with renal complications (Hariharan et al., 2000). Transplantation can cause various medical complications ranging from mild symptoms to life-threatening conditions (Adams, 2006). Herrington et al. (2019) revealed that cardiovascular disease (31% of deaths), infection (31%), and cancer (7%) were the leading causes of death following graft function in the first year after transplantation (Herrington et al., 2019). In the first year after kidney transplantation, the leading causes of death include cancer (29%), cardiovascular disease (23%), and infection (12%) (Hariharan et al., 2021). Furthermore, the high incidence of complications such as diarrhea, infection, and graft rejection after transplantation is a persistent challenge (Chong and Alegre, 2012; Mori et al., 2014). The administration of immunosuppressant drugs after transplantation, including tacrolimus and mycophenolate mofetil, is a primary strategy for preventing graft rejection (Tonshoff, 2020). It increases the likelihood of survival but concurrently results in a greater risk of complications after transplantation. Therefore, the development of new and feasible therapies that can attenuate the severity of complications after transplantation is necessary.

Contrary to expectations, substantial evidence has revealed that the adverse reactions observed after kidney transplantation are closely related to the gut microbiota of patients. The human body contains approximately 100 trillion microbial cells, including bacterial, archaeal, viral, and eukaryotic microbial communities, which directly inhabit the interface of the human body that is exposed to or connected to the external environment (Dominguez-Bello et al., 2019; Eng and Borenstein, 2019). Microorganisms play major roles in human health and can directly or indirectly affect human physiology in various ways (Dominguez-Bello et al., 2019). They can guide immune cells to rapidly and effectively respond to pathogenic invasion, simultaneously promote the completion of various metabolic functions, and affect most physiological functions *via* these basic functions (Gomaa, 2020).

**Figure 1:**
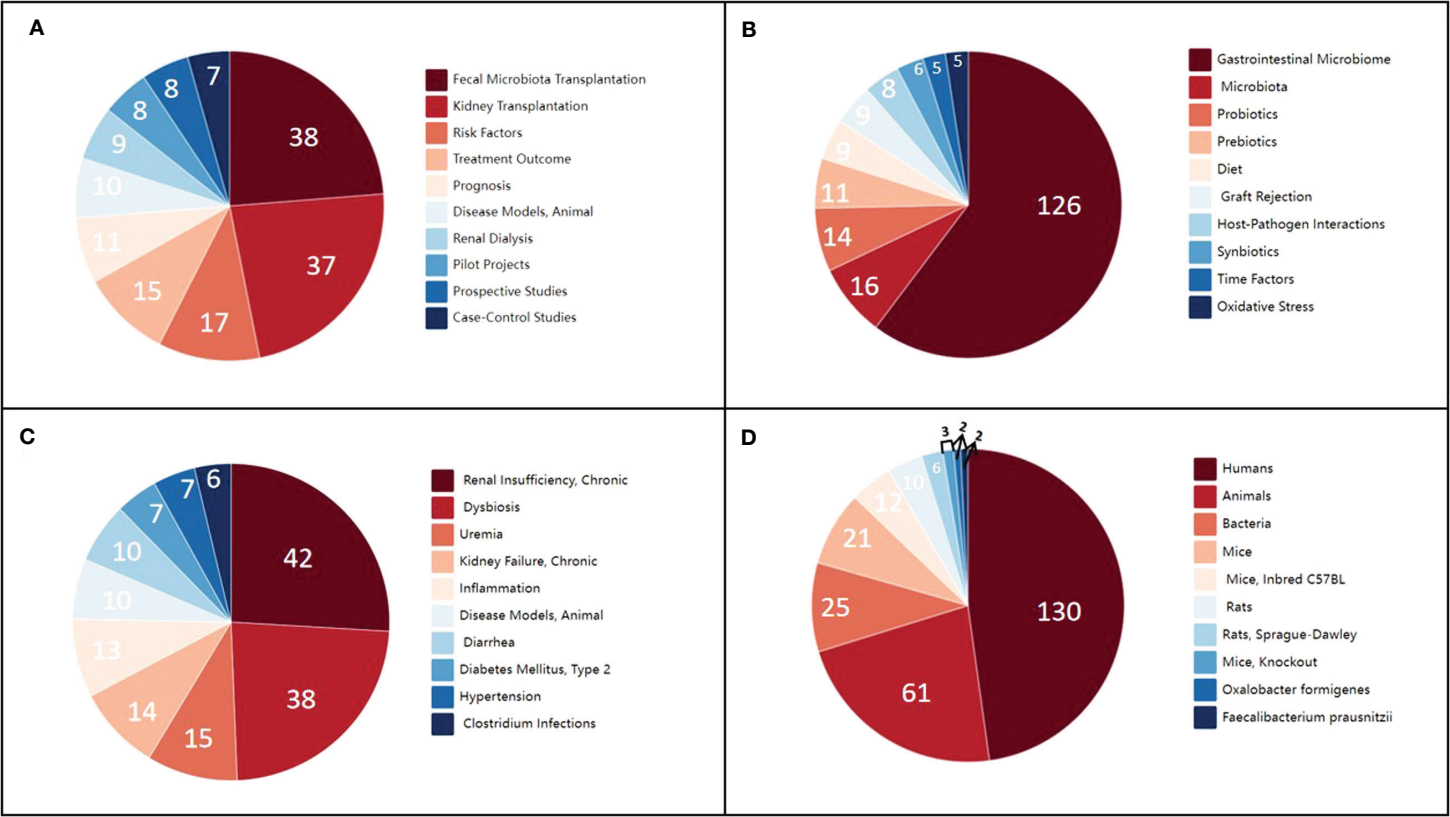
Clustering correlation between kidney transplantation and gut microbiota in published literature. **(A)** Analytical, diagnostic, and therapeutic techniques and equipment categories. **(B)** Phenomena and processes categories. **(C)** Diseases category. **(D)** Organisms category.

Recently, it has been reported that gut microbiota dysregulation adversely affects the prognoses of patients undergoing kidney transplantation (Xu et al., 2017). Increasing evidence has indicated the presence of a bidirectional relationship between gut microbes and nephropathy (Anders et al., 2013; Rukavina et al., 2020). Gut microbes aid in rebuilding the host–microbiome symbiosis, which can help alleviate kidney disease to a certain extent (Al Khodor and Shatat, 2017). An increasing number of scholars and research teams are investigating the relationship between gut microbiota and complications after kidney transplantation, refining the research on this topic (**Figure 1**). However, current research and reviews have focused only on specific types of complications. In this article, we discuss the evidence regarding gut microbiota from a more comprehensive perspective to evaluate the relationship between gut microbiota and various kidney-related complications.

## 2 The healthy profile of gut microbiota and kidney transplantation

The gut microbiome is an important but “invisible organ” in humans (Khanna and Tosh, 2014). Although this “organ” is not composed of actual organs, it is distributed throughout the body, with the intestine being the primary point of distribution (Gomaa, 2020). The total length of the gut is 175 m, leading to differences in the geographical distribution of gut microbiota in humans. The most abundant bacteria in the human gut are Firmicutes, *Escherichia*, and Bacteroidetes, which account for 95% of the total normal flora in healthy individuals; the remaining 5% include *Clostridium*, *Actinobacteria*, and other microbiota (Eckburg et al., 2005). Gut microbiota are among the most important components of the body and are related to various physiological conditions.

Gut microbiota, widely known as the mainstay of the digestive system in the body, were once considered to constitute an invisible organ with a metabolic capacity exceeding that of the liver (Nallu et al., 2017). In glycolipid metabolism, gut microbiota produce metabolites of short-chain fatty acids (colonic fiber fermentation), including acetate, propionate, and butyrate (Cummings et al., 1987), which positively contribute to the health and functioning of various systems. Along with mediating metabolic functions, the gut and microbiota, which collectively function as endogenous organs, play various physiological roles in the host, such as roles in liver damage, cardiovascular disease, sleep regulation, and circadian rhythms (Matenchuk et al., 2020), forming the microbe–gut–brain axis (Quigley, 2017), which maintains intestinal mucosal immunity (Shi et al., 2017). This suggests that gut microbiota dysbiosis is not only associated with digestive system diseases but also with other systemic diseases, such as obesity (Abenavoli et al., 2019), diabetes (Wu et al., 2020), and cardiovascular diseases (Witkowski et al., 2020). Winichakoon et al. (2022) comprehensively summarized and discussed major findings from *in vivo* and clinical data pertaining to the effects of gut microbiota on kidney transplantation (Winichakoon et al., 2022).

Kidney transplantation has been the gold standard renal replacement therapy for end-stage renal disease (ESRD) because it leads to superior survival and quality of life among patients compared with other replacement therapies (Wolfe et al., 1999). This has been consistently reported since the first successful kidney transplantation between identical twins in the 1950s (Force and Andreu, 2005). Transplantation and dialysis are the primary treatments for ESRD and these two treatments can significantly prolong a patient’s lifespan and improve their quality of life. According to statistics, patients aged 20–39 years survived for 8 years on dialysis and 25 years after transplantation (Augustine, 2018). In the United States, the 5-year survival rates of patients who underwent primary kidney transplantations from deceased and living donors were 72% and 85% (Hariharan et al., 2021), respectively, which were slightly different from those in Australia (81% and 90%), Europe (79% and 87%), and Canada (81% and 91%) (Wang et al., 2016). Compared to dialysis, kidney transplantation not only increases life expectancy but also improves the quality of life of several patients with ESRD. It also reduces the costs incurred due to other chronic treatments.

As noted earlier, after kidney transplantation, patients suffer from complications, such as cardiovascular disease (31% of deaths), infections (31% of deaths), and cancer (7% of deaths) (Hariharan et al., 2021). To reduce complications, patients undergoing kidney transplantation are chronically administered immunosuppressive agents to suppress the immune response after allogeneic kidney implantation. This inhibitory effect and the body’s autoimmune response to constantly reject foreign bodies are key determinants of transplantation (Ranganathan et al., 2006). Although these complications are not life-threatening, they greatly reduce the patients’ quality of life and affect their prognostic statuses. These complications differ from acute rejection, viral infection, cancer, and other uncontrollable risk factors and are closely related to the body’s immunity. A delicate balance exists between the two, and if this balance is disrupted because of other factors, patients undergoing kidney transplantation experience postoperative complications including infection and transplant rejection. Studies have revealed that the homeostasis of the gut microbiota significantly influences the immune system and can be used to alter the prognoses of patients undergoing kidney transplantation (Gioco et al., 2020).

## 3 Gut microbiota profiles of patients after kidney transplantation

The effects of dysbiosis that activate human immune responses, allograft rejection, and the pharmacokinetics of immunosuppressive drugs remain largely unknown (Anders et al., 2013) and may result in certain complications after transplantation such as the risk of infection (urinary infections and infectious diarrhea), adverse immune phenomena (autoimmune hemolytic anemia), transplant rejection, and increased mortality. Human and mouse studies have revealed that the abundance of the gut microbiota is significantly inversely associated with transplant prognosis, the abundance of microbial species, the number of donors and recipients, the estimated glomerular filtration rate within 6 months of transplantation, and species-differentiated microbiota are associated with an increased frequency of infection-related complications after transplantation. Thus, the presence of certain species in the gut before kidney transplantation is significantly associated with subsequent rejection. Therefore, the microbiota can affect the quality of life of patients after kidney transplantation (Ranganathan et al., 2006). Winichakoon et al. (2022) reported that the microbiota of an individual could alter the immune response of organ transplantation hosts *via* specific signaling pathways, such as the Myd88 and TLR9 pathways (Winichakoon et al., 2022).

Kim et al. (2020) reported that differences in the donor–recipient microbial community before transplantation can affect the function of early allografts, and this relationship can affect the incidence of infection within 6 months of transplantation. In genetically unrelated donor transplantations, unrelated individuals exhibited a more pronounced correlation between graft function and microbial similarity (Kim et al., 2020). Kim et al. (2020) suggested that the effects of unknown environmental and traditional genetic factors on the microbial community should be considered during post-transplantation management. Therefore, future studies investigating the relationship between gut microbiota and organ transplantation should consider sex, age, and ethnicity. Therefore, sex, age, and race should be considered when investigating the association between gut microbiota and organ transplantation.

Interestingly, we also found that various types of specific bacterial species were responsible for dysbiosis in patients undergoing kidney transplantation. Xiao et al. (2018) revealed that in densely populated areas in a multidimensional space composed of the same community (Xiao et al., 2018), the type and abundance of the human gut microbiota were similar. Arumugam et al. (2011) named this gut microbial composition “enterotype” (Arumugam et al., 2011; Costea et al., 2018) and found that its abundance was related to the age, sex, cultural background, and geographic location of individuals. In a study cohort, Costea et al. (2018) categorized enterotypes into three types and found that *Bacteroidetes* were dominant in Enterotype 1, *Prevotella* in Enterotype 2, and *Ruminococcus* in Enterotype 3. These intestinal types directly affect intestinal homeostasis and are closely associated with various acute and chronic diseases. In summary, based on this theory, personalized treatment should be administered in different populations; that is, different treatment methods should be selected for patients with different intestinal types.

Notably, a negative relationship exists between gut microbiota and the kidney. After drugs modulate the gut microbiota, kidney disease progression significantly slows down. When probiotics such as *Bacillus pasteuri* and *Lactobacillus sporogenes* were used to treat a rat model of chronic kidney disease (CKD), blood urea nitrogen (BUN) levels significantly reduced (Ranganathan et al., 2005), and the use of urease-positive *Pasteurella* reduced intestinal BUN levels. The concentration of urea, which also improves urinary protein excretion, reduces the glomerular sclerosis index (Ranganathan et al., 2006; Yoshifuji et al., 2016). Based on the findings of clinical studies summarized in **Table 1**, gut microbiota profiles differ considerably based on the pre- and post-transplantation status, as observed in 16S ribosomal ribonucleic acid polymerase chain reaction experiments.

**Table 1:** Clinical studies on the profiles of gut microbiota in patients after kidney transplantation.

Reference	Population	Post-transplantation phyla	Outcomes
Kim et al. (2020)	67 KTRs/67 donors;	↓Prevotellaceae; ↓*Prevotella*	6-month allograft function
Lee et al. (2015)	19 KTRs/54.7 Compared with the baseline	↑*Firmicutes*	↑ *Faecalibacterium prausnitzii*
Lee et al. (2019a)	168 KTRs/54healthy	↓Faecalibacterium prausnitzii; Eubacterium dolichum; Coprococcuscomes; Eubacteriumrectale	↓ butyrate-producing gut microbiota
Swate et al. (2020)	139 KTRs/105 healthy	↑Actinobacteria,*Firmicutes*; *Proteobacteria*	↓ butyrate-producing bacteria
Fricke et al. (2014)	60 KTRs/58 Compared with the baseline	↑*Actinobacteria; Bacteroidetes; Firmicutes; Proteobacteria*	↑Cr; ↑Actinobacteria (*Bifidobacterales*) ↑ Firmicutes (*Peptostreptococcus*)
Zaza et al. (2017)	20 KTRs/62.3 Compared with the baseline	↑*Actinobacteria*; *Firmicutes*; *Proteobacteria*	EVE + MMF (↑ msrA); TAC + MMF(↑ fliNY, pilM)
Guirong et al. (2018)	16 KTRs/84 CKD/53 healthy	↑*Bacteroidetes; Proteobacteria*; ↓*Firmicutes*	↑ Metabolism of (carbohydrates/amino acids/xenobiotics) ↓Metabolism of (cofactors/vitamins/nucleotides/terpenoids)
Chan et al. (2021)	12 KTRs/51healthy; 12 donors/56healthy	↓*Firmicutes*_G; Acutalibacteracae familyRikenellaceae family; ↑*Firmicutes*_A; Roseburia intestinalis Faecalibacterium prausnitzii D	gut microbiota richness, diversity, composition, and functional parameters in kidney transplant recipients
Lecronier et al. (2020)	19 Controls/ 15 NODAT/ 16 Diabetic	↑*Lactobacillus sp.;*↓Faecalibacterium prausnitzii*;* A. muciniphila in patients with diabetes	Higher percentage of *Lactobacillus* sp. carriage, higher relative abundance of *Lactobacillus* sp., and lower relative abundance of *A. muciniphila*.

KTR, kidney transplantation recipient; CKD, chronic kidney disease; EVE, everolimus; MMF, mycophenolate mofetil; mrsA, macrolide transport system; fliNY, flagellar motor switch protein; pilM, pilus assembly protein; “↓”, an upward trend in the abundance of gut microbiota and “↑”, a downward trend in the abundance of gut microbiota.

## 4 Kidney transplantation and regulation of gut microbiota

**Figure 2:**
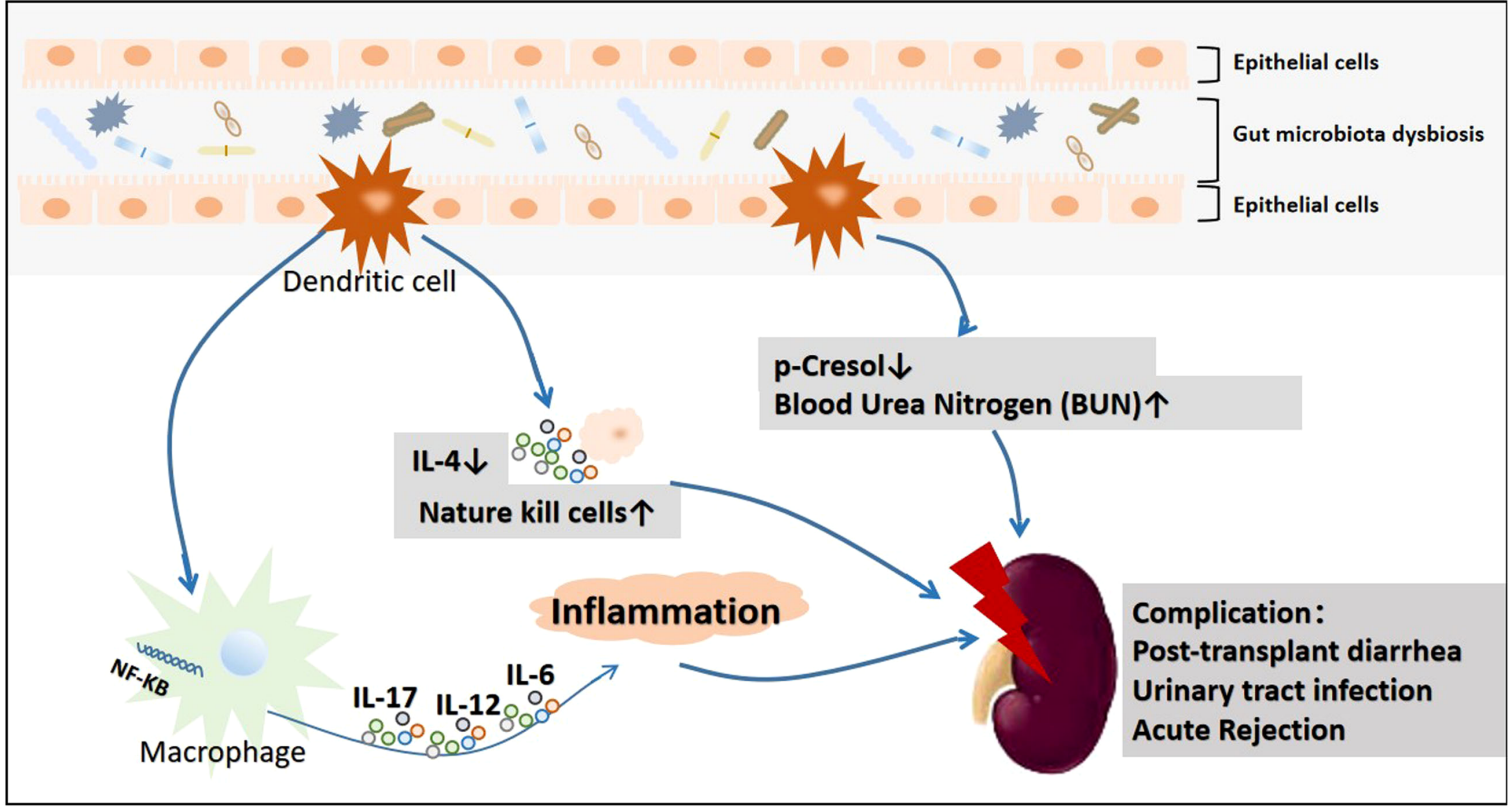
The primary mechanism underlying kidney damage after gut microbiota disorders.

As early as 2009, Jang et al. (2009) used mice to confirm the important role of the microecology of gut microbiota in kidney disease. They revealed that germ-free mice bred *via* technical means could be used to monitor *in vivo* abnormalities under the same exposure. A large population of killer T cells and extremely low concentrations of interleukin (IL)-4 were observed. However, after the gut microbiota were reintroduced to establish a stable gut microbiome, the conditions of the mice showed significant improvement (**Figure 2**). Accumulating evidence suggests that after gut microbiota dysbiosis affects the intestinal mucosa and causes intestinal inflammation, it eventually causes systemic inflammation by activating the nuclear factor (NF)-κB pathway (Anders et al., 2013). These findings provide a basis for the existence of the gut–immune axis *in vivo*. In view of the findings of these studies, a method to rebuild the intestinal microbiota and stabilize intestinal microecology can be developed, which can be innovatively used for the prevention and treatment of kidney diseases.

When gut microbiota are disturbed, inflammatory reactions can be activated *via* the NF-KB pathway (Anders et al., 2013), the degree of cresol and IL-4 (Jang et al., 2009)in intestinal mucosa can be reduced, and the degree of urea nitrogen can be increased (Ranganathan et al., 2005), which will lead to kidney damage.

The presence of multiple factors in patients before, during, and/or after kidney transplantation can result in changes in the type and quantity of the microbiota, thereby destabilizing it (Jang et al., 2009; Yoshifuji et al., 2016). Some scientists have revealed that the gut microbiota in patients undergoing kidney transplantation who have undergone fecal microbiota transplantation (FMT) are highly similar to the type composition of the donor’s gut microbiota and will tend to gradually normalize (Ianiro et al., 2020). Wang et al. (2021a) revealed that in kidney transplantation recipients (RTRs), the dominant flora (Prevotella_9, relative abundance 35.9%) (Wang et al., 2021a) was *Bacteroides* (relative abundance 60.6%) after 2 months of FMT administration.

A plethora of evidence supports the idea that gut microbiota dysbiosis plays a vital role in the outcomes of organ transplantation, especially kidney transplantation. Modification of the gut microbiota in response to routine therapeutic approaches, such as the administration of immunosuppressive drugs and prophylactic antibiotics post transplantation (Kim and Song, 2020), may adversely affect graft outcomes, thus leading to graft rejection, infection, fibrosis, and alterations in drug metabolism. As summarized in **Table 2**, prebiotics, synbiotics, and FMT can transform a state of gut dysbiosis into colonization by healthy gut microbiota.

**Table 2:** Effects of probiotics or synbiotics on kidney transplantation outcomes.

Reference	Population (mean ages)	Post-transplantationphyla	Outcomes
Wang et al. (2021a)	A 37-year-old woman with CRKP infection at 1 month after kidney transplantation	↑*Phascolarctobacterium* and *Lachnoclostridium*; ↓*Klebsiella*	Fecal microbiota transplantation (FMT) to treat an infection caused by CRKP for a patient undergoing kidney transplantation.
Biehl et al. (2018)	A 50-year-old woman undergoing immunosuppressive treatment after kidney transplantation 3 years ago	↓*Enterobacteriaceae*; ↑ classes of bacilli and clostridia	FMT for recurrent urinary tract infections.
Guida et al. (2017)	36 KTRs(mean age: 49.6 years) with transplantation vintage > 12 months	↓plasma p-Cresol	Treatment with synbiotics may be effective for lowering the plasma p-Cresol levels in KTRs.
Chan et al. (2022)	27peoplein the intervention group(mean age:52.9 years);29 peoplein the control group(mean age: 54.7 years)	Median GSRS score change −0.15/−0.28/−0.07	Prebiotics significantly reduced gastro- intestinal symptoms.
Lin et al. (2018)	A 64-year-old woman, transplantation (2002); A 34-year-old woman, transplantation (2013); A 62-year-old woman, transplantation (2000)	With the resolution of diarrheal symptoms; no further CDI recurrence observed over 10 months of follow-up	Detailed FMT protocol and experience in treating a series of patients with SOT with rCDI.
Wei et al. (2019)	A 32-year-old Chinese woman, who developed DIHS-associated MODS, underwent FMT four times at a frequency of once every 6 days. The healthy control was a 23-year-old man who was a graduate student.	↑*Lachnospiraceae, Ruminococcaceae*, *Veillonellaceae* ↓ *Proteobacteria*, *Enterobacteriaceae*, and *Alcaligenaceae families*. At the genus level:↑ *Roseburia*, *Prevotella*, *Bacteroides*, *Oscillospira*, and *Faecalibacterium*	FMT is effective for treating intestinal failure associated with DIHS.

KTR, kidney transplantation recipient; CKD, chronic kidney disease; CRKP, carbapenem-resistant Klebsiellapneumoniae; FMT, fecal microbiota transplantation; CDI, Clostridium difficile infection; SOT, solid organ transplantation; GSRS, gastrointestinal symptom rating scale; DIHS, drug-induced hypersensitivity syndrome; “↓”, an upward trend in the abundance of gut microbiota and “↑”, a downward trend in the abundance of gut microbiota.

**Figure 3:**
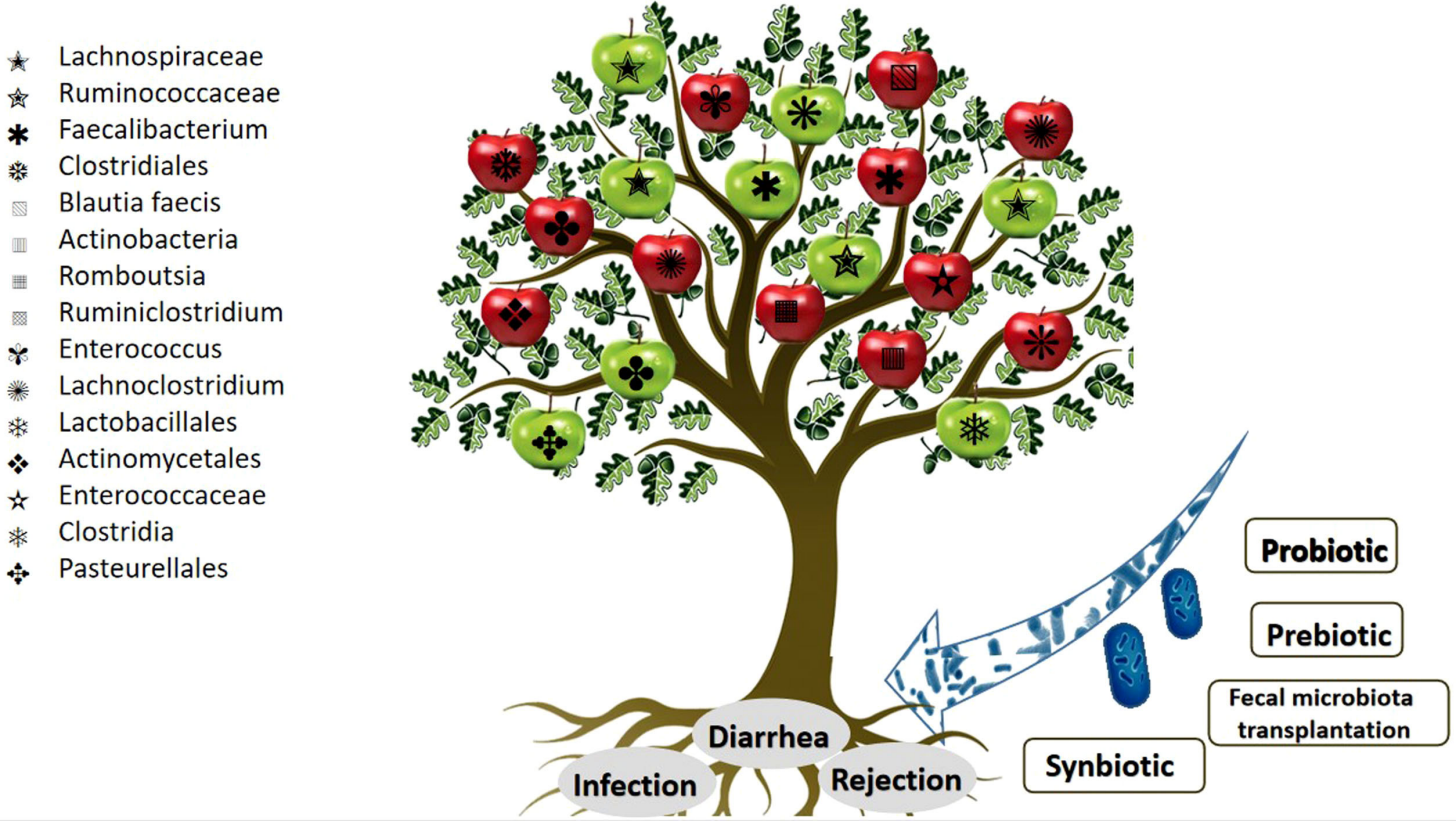
Schematic view of the link between gut microbiota and kidney transplantation complications. The red apples represent an upward trend in the abundance of gut microbiota. The green apples represent a downward trend in the abundance of gut microbiota.

According to Guida et al. (2017), synbiotics can effectively lower plasma p-cresol levels in patients undergoing kidney transplantation. P-cresol generally accumulates when its production by the dysbiotic gut microbiome increases (Guida et al., 2017). In a study, 27 recipients of kidney transplantation with gastrointestinal symptoms received a prebiotic powder suspension with breakfast for 7 weeks (Chan et al., 2022). In this randomized placebo-controlled trial, prebiotics significantly suppressed gastrointestinal symptoms. In another study by Lin, three RTRs showed no further CDI recurrence or diarrheal symptoms during the follow-up period after receiving FMT treatment *via* colonoscopy (Lin et al., 2018) (**Figure 3**).

Furthermore, clinical studies have revealed that probiotic supplementation effectively improves kidney function. Ranganathan et al. (2010) and other researchers administered probiotic strains such as *Lactobacillus acidophilus*, *Lactobacillus acidophilus*, and *Streptococcus thermophilus* to 46 patients with CKD III or IV and found that their renal function and quality of life significantly improved after probiotic intake for 6 months (Ranganathan et al., 2010). Grosen et al. (2019) reported a case wherein a patient suffered from urinary tract infections (UTIs) caused by broad-spectrum lactamase-producing (ESBL+) *Klebsiella pneumoniae* but showed no symptoms of UTI after FMT treatment (Grosen et al., 2019). In 2021, Wang et al. (2021a) presented a case report where carbapenem-resistant *Klebsiella pneumoniae* (CRKP) infection was treated using FMT in China (Wang et al., 2021a). After FMT, not only did the CRKP test of urine and anal swab cultures yield negative results but a greater relative abundance of *Phascolarctobacterium* and *Lachnoclostridium*was noted along with the depletion of *Klebsiella*.

Kim reported that gut microbiota profiles considerably vary between recipients and donors (Kim et al., 2020). Recipients exhibited lower levels of *Prevotellaceae* and *Prevotella*, whereas the opposite was observed in donors. Lee et al. (2015) revealed that in patients after kidney transplantation, a lower diversity of Firmicuteswas observed (Lee et al., 2015). Lee et al. (2019a) also reported lower relative abundances of *Faecalibacterium prausnitzii*, *Holdemanella biformis*, *Eubacterium dolichum*, *Coprococcus comes*, *Subdoligranulum variabile*, and *Eubacterium rectale (* Lee et al., 2019a). Swarte et al. (2020) reported increased proportions of Firmicutes and Proteobacteria in 139 kidney transplant recipients and 105 healthy controls (Swarte et al., 2020). Fricke et al. (2014) reported that increased *Actinobacteria* and Firmicutes abundance is associated with an increased risk of graft dysfunction, as identified by an increase in chromium concentration after kidney transplantation (Fricke et al., 2014). Zaza et al. (2017) revealed increased abundances of Actinobacteria, Firmicutes, and Proteobacteria in patients who were administered different immunosuppressive drug regimens after kidney transplantation (Zaza et al., 2017). Contrary to expectations, Guirong et al. (2018) reported a decrease in Firmicutes abundance instead of an increase in recipients of kidney transplants (Guirong et al., 2018). Chan et al. (2021) classified Firmicutes into two types and reported a significantly greater abundance of Firmicutes A and a relatively lower abundance of Firmicutes G (Chan et al., 2021). This may explain the contradictory results reported by different studies.

## 5 Kidney transplantation outcomes and profiles of gut microbiota

### 5.1 Diarrhea after transplantation

Diarrhea after transplantation is a common complication observed after solid organ transplantation (Zhang et al., 2021), and a large registry analysis identified that diarrhea after transplantation negatively affects graft and patient survival (Aulagnon et al., 2014). Diarrhea is generally considered to exert a mild impact on patients’ quality of life, but severe episodes have a significant impact on drug metabolism and the prognoses and survival of patients (Zhang et al., 2021), such as a drastic decline in the functionality of the transplanted kidney or a significant increase in patient mortality (Ekberg et al., 2007). According to Medicare claims registered with the United Network for Organ Sharing, diarrhea occurs in 20%–50% of solid organ transplant recipients in the first year after transplantation (Angarone and Snydman, 2019), with an incidence of diarrhea after kidney transplantation of 11.5% and the cumulative incidences of diarrhea at 2 and 3 years of 17.5% and 22.6% (Bunnapradist et al., 2008), respectively.

Along with certain dietary elements, antibiotic use, the induction of transplant-specific lymphatic failure (Li et al., 2013), and immune rejection also contribute to an imbalance in the gut microbiota. Some studies have revealed that only a few patients who harbored gastrointestinal pathogens during transplantation developed diarrhea after transplantation, and only a few patients exhibited the same pathogenic flora colonizing the gastrointestinal tract before and after transplantation (Westblade et al., 2019). Diarrhea can be induced by various factors. Bunnapradist et al. (2006) showed that tacrolimus and mycophenolate mofetil increase the risk of noninfectious diarrhea, graft loss, and patient death (Bunnapradist et al., 2006). Therefore, pathogens that cause diarrhea in the gastrointestinal tract are more likely to colonize and cause chronic infections in the body, forging a relationship with the progression of diarrhea after transplantation. Study findings have further indicated that gut microbiota can be isolated from diarrheal stool samples, with reduced microbial diversity and decreased abundance of commensal bacterial species. Most cases of diarrhea after transplantation are different from common infectious diarrhea, which is related to intestine-related dysbiosis. Patients undergoing kidney transplantation show a greater abundance of *Proteobacteria* than healthy individuals (Arslan et al., 2007). After kidney transplantation, patients are more susceptible to opportunistic pathogens than immunocompromised healthy individuals (Aulagnon et al., 2014).

Interestingly, Westblade et al. (2019) revealed that in the first 3 months after transplantation, 47 (33%) of 142 kidney transplant recipients developed diarrhea after transplantation, of which 24 recipients exhibited extensive microbial colonization (Westblade et al., 2019). Lee et al. (2014) reported differences in the diversity of the gut microbiota between healthy individuals and kidney transplant recipients. The latter exhibited an increase in *Enterococcus*, *Escherichia*, and *Lachnoclostridium*and a decrease in *Eubacterium*, *Anaerostipes*, *Coprococcus*, *Romboutsia*, *Ruminococcus*, *Dorea*, *Faecalibacterium*, *Fusicatenibacter*, *Oscillibacter*, *Ruminiclostridium*, *Blautia*, *Bifidobacterium*, and *Bacteroides*. Lee et al. (2014) also reported that an increase in *Faecalibacterium* abundance was positively correlated with future tacrolimus dosing at 1 month (Lee et al., 2014). Swarte et al. (2020) reported an increasing trend in the abundance of *Proteobacteria* and a lower abundance of *Faecalibacterium prausnitzii*, *Gemmiger formicilis*, *Eubacterium rectale*, *Coprococcus catus*, Cop*rococcus comes*, and *Roseburia (* Swarte et al., 2020). This study also reported the depletion of butyrate-producing bacteria caused by antibiotics and immunosuppressive agents and this was considered to be associated with diarrhea after kidney transplant. Wei et al. (2019) reported increased abundance of *Lachnospiraceae*, *Ruminococcaceae*, and *Veillonellaceae*, and decreased abundance of Proteobacteria, *Enterobacteriaceae*, and *Alcaligenaceae* in a 32-year-old kidney transplant recipient. These findings reveal that butyrate-producing bacteria are lost in RTRs (Flint et al., 2015). Diarrhea after transplantation was not associated with common infectious diarrheal pathogens but with dysbiosis.

Norovirus has been attributed to approximately 25% of cases with diarrhea after transplantation. *Clostridium difficile* infection occurs in 2%–7% of kidney transplant recipients (Chan et al., 2022). Evidence from several studies has suggested that *C. difficile* also causes nosocomial infections that patients contract within the first few months after transplantation (Guida et al., 2017; Lin et al., 2018). Kujawa-Szewieczek et al. (2015) attempted to use *Lactobacillus plantarum* 299v as a preventive agent against this infection in patients receiving antibiotics and observed that probiotics could efficiently prevent *C. difficile* infection in RTRs (Kujawa-Szewieczek et al., 2015). Lee et al. (2019b) also reported that some patients continued to experience diarrhea despite repeated monitoring tests for *C. difficile* before FMT, which yielded negative results. The authors also revealed that the abundance of *Ruminococcus* significantly decreased in samples collected after the onset of diarrhea symptoms (Lee et al., 2019b) compared to samples collected before diarrhea. A summary of these findings is provided in **Table 3**.

**Table 3:** Association between gut microbiota and diarrhea in kidney transplantation recipients: evidence from clinical studies.

Reference	Population (mean ages)	Key findings	Outcomes
Swarte et al. (2020)	Fecal samples were collected from 139 KTRs (50% men, mean age: 58.3 ± 12.8 years) and 105 healthy controls (57% men, mean age: 59.2 years).	↑ ***Proteobacteria*,** ↓ *Actinobacteria* ↓ butyrate-producing bacteria	On comparing the gut microbiome of KTRs to healthy controls, KTRs were found to suffer from dysbiosis, a disruption in the balance in the gut microbiome.
Fricke et al. (2014)	Fecal samples were collected from 40 KTRs (63% men, mean age: 58 years).	↓*Proteobacteria, Escherichia*, *Porphyromonas* (urine), *Haemophilus*, *Neisseria*, *Pasteurella* (oral swabs)	Characteristics of the microbiota that can serve as diagnostic markers for transplant health and guide intervention strategies for improving transplantation outcomes.
Lee et al. (2014)	Fecal samples were collected from 26 KTRs (50% men, mean age: 56years).	↑*Faecalibacterium* in the dose escalation group	Distinct microbiota structures were observed in allograft recipients with post-transplant diarrhea, acute rejection, and *Enterococcus*UTI.
Lee et al. (2019b)	Fecal samples were collected from 71 KTRs: 25 from the diarrhea group (40% men, mean age: 56 years) and 46 from the no diarrhea group (59% men, mean age: 53 years).	↓*Eubacterium*, *Anaerostipes*, *Coprococcus*, *Romboutsia*, *Ruminococcus*, *Dorea*, *Faecalibacterium*,*Fusicatenibacter*, *Oscillibacter*, *Ruminiclostridium*, *Blautia*, *Bifidobacterium*, and *Bacteroides*; ↑*Enterococcus*, *Escherichia*, and *Lachnoclostridium*	Colonization with gastrointestinal pathogens, particularly *Clostridium difficile*, is commonly observed in patients after kidney transplantation but does not predict subsequent diarrhea.
Zhang et al. (2021)	Fecal samples were collected from 40 KTRs (42% men, mean age: 52 years) and 57 healthy controls (67% men, mean age: 56 years).	Suggesting fecal β-glucuronidase activity could be a novel biomarker for gastrointestinal tract-related MMF toxicity.	Fecal β-glucuronidase activity could be a novel biomarker for gastrointestinal tract-related MMF toxicity.

KTRs, kidney transplantation recipients; MMF, mycophenolate mofeti; “↓”, an upward trend in the abundance of gut microbiota and “↑”, a downward trend in the abundance of gut microbiota.

Modulation of the gut microbiota profile *via* FMT or administration of probiotics has positively affected RTRs. Aulagnon et al. (2014) reported the clinical characteristics of a patient with chronic diarrhea after kidney transplantation (Aulagnon et al., 2014). After Masson’s trichrome staining, the intracellular accumulation of calcium oxalate monohydrate was microscopically observed, and the deposition of massive crystals disrupted the tubular structure, thus inducing inflammation and fibrosis in the scarred area. Gu et al. (2018) showed that diarrhea symptoms in patients undergoing kidney transplantation improved after FMT (Gu et al., 2018). Zhang et al. (2021) recently revealed that fecal β-glucuronidase activity could serve as a novel biomarker for gastrointestinal tract-related mycophenolate mofetil toxicity.

### 5.2 UTI

UTI is an infection primarily caused by bacteria invading urothelial cells, inducing an immune response in the body to resist infection (Fiorentino et al., 2019). The incidence of kidney transplantation is considerably higher in patients with UTIs than in the general population (Alangaden et al., 2006; Rice and Safdar, 2009; Karuthu and Blumberg, 2012). Asymptomatic bacteriuria constitutes 44% of UTI cases, whereas uncomplicated and complicated UTI account for 32% and 24% of the cases, respectively. Patients undergoing kidney transplantation account for approximately 40%–50% of all infectious complications (Meena et al., 2021), with bacteriuria detectable in approximately 40% of patients within the first year (Magruder et al., 2019). Furthermore, nearly 50% of UTI cases occur within 3 years of transplantation. UTIs not only affect patients’ quality of life but also increase the risk of other complications in transplant recipients, which affect long-term graft survival and rejection (Castaneda et al., 2013; de Castro Rodrigues Ferreira et al., 2017).

Fiorante et al. (2010) reported that asymptomatic bacteriuria alone was associated with a seven-fold increased risk of pyelonephritis in a population of patients undergoing kidney transplantation (Fiorante et al., 2010), and each episode of UTI that occurred after the transplantation reduced graft function to some extent (Fiorentino et al., 2019). In a retrospective study, approximately 1%–3% of patients with UTIs developed bacteremia, and >20,000 kidney transplant recipients developed UTIs 6 months after transplantation (Lee et al., 2013), which significantly increased the risk of death and graft loss. Recent studies support the role of gut microbiota in UTI pathogenesis, with *Enterobacteriaceae* being the most common cause of UTI after transplantation (Lee et al., 2013). In a study of nontransplantation patients, children with *Escherichia coli* UTIs exhibited greater gut *E. coli* abundance than other children. In another experiment including kidney transplant recipients, the identification of urine strains revealed that the *E. coli* present in urine was genetically similar to the *E. coli* present in stool samples collected from the same participants, supporting the notion that gut microbiota dysbiosis is the primary source of UTIs (Magruder et al., 2020). Magruder et al. (2020) showed that RTRs with UTIs experienced the same shifts in microbial diversity, with an increased abundance of Firmicutes and Proteobacteria (Magruder et al., 2019; Magruder et al., 2020). A summary of these findings is provided in **Table 4**.

**Table 4:** Association between gut microbiota and urinary tract infection in kidney transplantation recipients: evidence from clinical studies.

Reference	Population (mean ages)	Key findings	Outcomes
Lee et al. (2014)	Fecal samples of 26 KTRs (50% men, mean age: 56 ± 8 years). The median time after transplantation of KTRs was 3 months.	↑ *Enterococcus* ↑ *Enterococcus* UTI compared to 0% in the 23 patients without *Enterococcus* UTI	Distinct microbiota structures were observed in allograft recipients with post-transplantation *Enterococcus* UTI.
Magruder et al. (2020)	Fecal samples were collected from 168 KTRs:51 in the Enterobacteriaceae bacteriuria group (27% men, mean age: 57 years) and 117 in the no Enterobacteriaceae bacteriuria group (67% men, mean age: 53 years). Median time after the transplantation of KTRs was 6 months.	↑*Faecalibacterium* and *Romboutsia*; ↓*Lactobacillus* in the no *Enterobacteriaceae* *bacteriuria* group	Bacterial taxa associated with a decreased risk for *Enterobacteriaceae* UTI in KTRs, which will help support future studies reporting the modulation of gut microbiota as a novel treatment for preventing UTIs.
Magruder et al. (2019)	Fecal specimens from 168 KTRs. Median time after the transplantation of KTRs was 3 months.	↑*Escherichia* in the *Escherichia bacteriuria* group than in the no *Escherichia bacteriuria* group; ↑*Enterococcus* in the *Enterococcus* bacteriuria group	The results support a gut microbiota–UTI axis, suggesting that the modulation of the gut microbiota may be a novel strategy for preventing UTIs.

KTRs, kidney transplantation recipients; UTI, urinary tract infection; “↓”, an upward trend in the abundance of gut microbiota and “↑”, a downward trend in the abundance of gut microbiota.

Interestingly, some patients with UTI after kidney transplantation show improvements in clinical symptoms and urinary sensation without antibiotics. A preliminary speculation may be related to the body’s response to acute infection. This implies that unconventional methods can be used to stabilize the type and abundance of microbiota. Contrary to expectations, Biehl reported that FMT (via frozen capsulized microbiota) improved UTI symptoms in kidney transplant recipients (Biehl et al., 2018).

Gram-negative bacteria are responsible for the occurrence of 70% of UTIs in RTRs, with *E. coli* accounting for 30%–80% of kidney transplantation-related UTIs (Valera et al., 2006; Pelle et al., 2007). *Klebsiella*, *Pseudomonas aeruginosa*, other gram-negative bacteria, and *Proteus* are common pathogens that cause UTIs. When gut microbiota invade the body to induce UTI or bacteriuria, the bacteria continuously invade the urothelium by producing virulence factors and specific adhesins on the surface of the bacterial membrane to stimulate urothelial cells, which produce pro-inflammatory factors, such as IL-8. These factors promote the migration of neutrophils to infected urothelial cells and facilitate inflammatory immune responses (Fiorentino et al., 2019). Modulation of gut microbiota may play a role in the treatment of UTI in RTRs.

### 5.3 Acute rejection

Thirty-nine percent of patients experience at least one acute rejection within 1 year of transplantation (Carron et al., 2019). Acute rejection can lead to graft failure within a short duration (Kidney Disease: Improving Global Outcomes (KDIGO) Transplant Work Group, 2009).It can also lead to a greater three-fold risk of graft glomerular disease and loss of graft function in patients within 1 year of transplantation than in healthy controls (Gloor et al., 2007). Gut microbiota have been associated with the occurrence of acute rejection, and the relative abundances of *Lactobacillus*, *Enterococcus*, anaerobic bacteria, and *Clostridium* termites were found to be higher in patients with acute rejection, whereas those of *Clostridium*, *Bacteroides*, *Cyanobacteria*, and *Lachnospira*were lower (Lee et al., 2014). Wang revealed that 29 recipients of kidney transplantation (including 24 acute rejection recipients) reported a risk of graft rejection in response to altered proportions of Firmicutes and Bacteroidetes compared to those of *Proteobacteria*, *Actinobacteria*, *Lactobacillales*, *Clostridia*, and *Faecalibacterium*, with the abundances of the former decreasing and that of the latter increasing (Wang et al., 2021b). Carron et al. (2019) reported that the concentrations of circulating sCD14 and intestinal fatty acid-binding protein (iFABP) inversely correlated with a stronger prompt for acute rejection after transplantation in 788 recipients of kidney transplantation (Carron et al., 2019). A summary of these findings is provided in **Table 5**.

**Table 5:** Association between gut microbiota and acute rejection in kidney transplantation recipients: evidence from clinical studies.

Reference	Population (mean age)	Key findings	Outcomes
Lee et al. (2014)	Fecal samples of 26 KTRs (50% men, mean age: 56 ± 8 years). Median time after transplantation of KTRs was 3 months.	↓*Clostridiales*,*Bacteroidales*,*Ruminococcus*, *Bacteroides*,*Lachnospiraceae,Blautia, Eubacterium dolichum;* ↑*Lactobacillales*, *Enterococcus*, *Anaerofilum*, and *Clostridium tertium*.	Distinct microbiota structures were observed in allograft recipients with post-transplantation *Enterococcus* UTI.
Wang et al. (2021b)	Fecal samples were collected from 24 ARs (mean age: 35 years) and 29 KTRs (mean age: 38 years). The median time after the transplantation of KTRs was 3 months.	↓ Firmicutes, Bacteroidetes; ↑*Proteobacteria*, *Actinobacteria*, *Lacto- bacillales*, *Clostridia*, *Clostridiales*, and *Faecalibacterium*	Provide a foundation for further investigation on the role of gut microbiota in AR after kidney transplantation.
Carron et al. (2019)	Fecal samples of 934 individuals were collected: 146 study participants (mean age: 50 years) and 788 KTRs (mean age: 52.4 years). The median time after the transplantation of KTRs was 3 months.	The circulating sCD14 concentrations decreased significantly at 1 year after transplantation; significant decrease in biologically active LPS; circulating iFABP concentrations decreased significantly at 1 year after transplantation; circulating iFABP concentrations at 1 year after transplantation were inversely correlated with the GFR	Individuals with higher pre-transplantation sCD14 levels are less likely to develop AR after transplantation

“↓”, an upward trend in the abundance of gut microbiota and “↑”, a downward trend in the abundance of gut microbiota.

Some scholars have studied how the gut microbiota can cause acute rejection and proposed that if patients with ESRD maintain a stable total lipopolysaccharide (LPS) concentration for a long duration and a state of low LPS activity, the level of inflammatory biomarkers in patients undergoing kidney transplantation can be improved 1 year after transplantation. This could help protect patients who have undergone kidney transplantation from acute rejection (Carron et al., 2019). Authors have also proposed the observation of an intestinal epithelial cell damage marker, iFABP (Piton and Capellier, 2016), to assess the integrity of the intestinal epithelial barrier and the measurement of inflammatory biomarkers (sCD14 and cytokines) concurrently to explore the pathways and mechanisms underlying LPS elimination (Lee et al., 2014) and body-induced acute immune rejection.

## 6 Conclusion and perspectives

The gut microbiota exerts certain regulatory effects on various organ systems, and kidney-related diseases are no exception. An increasing number of individuals are now focusing on the bidirectional relationship between gut microbiota and kidney transplantation complications. Low kidney function can result in intestinal microbiota imbalance, and an imbalance in the microecological environment can further accelerate the progression of complications in kidney transplant recipients. However, prophylactic drugs can be administered to regulate the microbiota of patients undergoing kidney transplantation to reduce the occurrence and progression of subsequent complications. However, this question does not yet have a definitive answer.

The results of the human microbiome project are still being reported, and the roles of treatments with prebiotics and gut microbiota transplantation in human health and disease prevention remain unclear. However, it is conceivable that in the near future, we will use probiotics or other drugs that regulate gut microecology instead of traditional drugs that exert significant adverse effects. A clinical team used probiotics for the postoperative treatment of patients undergoing kidney transplantation and reported a significant decrease in the incidence of diarrhea and intestinal complications. However, the number of test groups was small, and the results are not generally representative. More basic clinical studies are required to explore this topic.

It is necessary to evaluate whether this preventive treatment can accurately control the patient’s condition and reduce the occurrence and progression of complications, and whether early microorganisms in the body interact with drugs including antibiotics and prebiotics. Conversely, *Clostridiales* has been reported as a potential mechanistic biomarker in different irritable bowel syndrome subtypes and represents a potential therapeutic target. We also identified a potential mechanistic biomarker that could be used as an early monitoring indicator of postoperative complications or disease development in patients undergoing kidney transplantation.

## Author contributions

JXY, FFH, JS, ZZ, YMW and JJY drafted the manuscript, JXY, FFH, JS and ZZ wrote the manuscript. All authors read, edited and approved the final manuscript.
